# Absconding among admitted patients with bipolar affective disorder diagnosis in Uganda

**DOI:** 10.1186/s12888-023-04794-w

**Published:** 2023-05-04

**Authors:** Joan Abaatyo, Alain Favina, Mark Mohan Kaggwa

**Affiliations:** 1 Department of Psychiatry, Faculty of Medicine, Mbarara University of Science and Technology, Mbarara, Uganda; 2 Department of Psychiatry and Behavioral Neurosciences, McMaster University, Ontario, ON Canada

**Keywords:** Bipolar affective disorder, Absconding, cannabis, Mood lability, Psychotherapy, Haloperidol

## Abstract

**Background:**

Hospitalization is often necessary for individuals with Bipolar affective Disorder (BAD) during severe manic or depressive episodes, as well as for stabilizing treatment regimens. However, a significant proportion of patients admitted for treatment of BAD abscond or leave the hospital without permission during their stay. In addition, patients managed for BAD may have unique characteristics that might force them into absconding. For example, the high prevalence of co-morbid substance use disorder – craving to use substances, suicidal behaviors – attempts to die by suicide, and cluster B personality disorders – characterized by impulsive acts. It is, therefore, essential to understand the factors contributing to absconding among patients with BAD, to facilitate designing strategies for preventing and managing this behavior.

**Method:**

This study was based on a retrospective chart review of the inpatients diagnosed with BAD at a tertiary psychiatry facility in Uganda from January 2018 to December 2021.

**Results:**

Approximately 7.8% of those with BAD absconded from the hospital. The likelihood of absconding among those with BAD increased with the use of cannabis [adjusted odds ratio (aOR) = 4.00, 95% confidence interval (CI) = 1.22–13.09, p-value = 0.022] and having mood lability [aOR = 2.15, 95% CI = 1.10–4.21, *p*-value = 0.025]. However, receiving psychotherapy during the admission (aOR = 0.44, 95 CI = 0.26–0.74, *p*-value = 0.002) and treatment with haloperidol (aOR = 0.39, 95% CI = 0.18–0.83, *p*-value = 0.014) reduced the likelihood of absconding.

**Conclusion:**

Absconding among patients with BAD is common in Uganda. Those with symptoms of affective lability and those with comorbid cannabis use tend to abscond more, while those who receive haloperidol and psychotherapy are less likely to abscond.

## Introduction

Bipolar affective disorder (BAD) is a chronic mental health condition characterized by episodes of mania and depression. Hospitalization is often necessary for individuals with BAD during severe manic or depressive episodes, as well as for stabilizing treatment regimens [1]. However, a significant proportion of patients admitted for treatment of bipolar disorder abscond or leave the hospital without permission during their stay [2].

Absconding can have severe consequences for the patient, including worsening their condition and an increased risk of harm to themselves or others [3–6]. For example, exposure to violence, suicide, homicide, poor control of symptoms, loss of contact and confidence with psychiatric services, and potential legal liability for the hospital [3, 4]. In addition to the above effects of absconding, high suicide rates between 20% and 30% of patients have been reported [7, 8]. While most absconds slip away with no harm caused, and return by themselves, usually within 24 h, they still cause the staff an enormous amount of anxiety [9, 10]. Additionally, the confidence of the relatives of the absconds can be severely damaged [11].

The phenomenon of absconding has been associated with various predictors, which include younger age, male sex, single status, shorter length of hospital stays, personality disorders, substance use disorders, patients who have been referred to the psychiatric hospital by police, those who are unemployed [12–14]. Various clinical factors have also been associated with absconds, for example, a history of absconding behaviors, substance misuse, or dependence [6]. A history of non-compliance with treatment, a history of sexually inappropriate behavior, and a history of childhood conduct problems and current symptom presentation including pacing, appearing to be hyper-vigilant, severely distressed, agitated, tormented, expressing suicidal ideation, or simply requesting go to the shops [3]. Other predictive factors include the patient’s level of functioning and ability to comprehend, understand and comply with the admission process [3]. Patients managed for BAD may have special characteristics that might force them into absconding such as impulsive acts. It is therefore important to understand the factors contributing to absconding among patients with BAD, to facilitate designing strategies for preventing and managing this behavior.

This study provides a different lens to the literature since most previous studies about absconding have focused on Schizophrenia, forensic patients, or psychiatric patients in general [9, 15, 16]. In addition, the study was conducted in Africa, where the annual rate of absconding from psychiatry units is highest; for example, In Uganda alone, about 10 to 50 patients are estimated to abscond every month [17–19].

## Methods

This study was based on a retrospective chart review of the inpatient diagnosed with BAD at a tertiary psychiatry facility in Uganda from January 2018 to December 2021. Retrospective chart reviews have previously been used to understand various mental health conditions in the country [20–22]. Based on their diagnosis, the patients were identified from a database of all patients admitted at Mbarara Regional Referral Hospital psychiatry unit between 2018 and 2021. Census sampling method was used in which all files of patients with BAD admitted from January 2018 and December 2021. No file was excluded since all files had captured age and gender. The formal consent for the data of the included patients was waivered by the Mbarara University of Science and Technology Research Ethics Committee since the data was retrospective and we could not trace all involved participants (Ethical approval number: MUSTREC-2021-229). However, the following information was captured for this study (i) age, (ii) gender, (iii) substances of addiction used, (iv) clinical symptoms, (v) abscondment or discharge from ward, (vi) diagnosis, (vii) family history of mental illness, (viii) employment status, (ix) number of days with symptoms before admission, and (x) number of days on the ward.

### Data analysis

Data were analyzed using STATA version 15.0. Categorical variables were presented with frequencies and percentages. The Gaussian assumption was used to assess normality based on the Shapiro-Wilks test and histograms. Skewed continuous variables were presented in median and interquartile ranges. Chi-square tests for categorical variables or rank sum tests for continuous variables were performed to determine significant differences between individuals with a diagnosis of BAD who absconded from the hospital and those who did not. Statistical significance values were included in logistic regression analysis using absconding as the dependent variable. Factors significant at bivariate logistic analysis were tested for collinearity using variance inflation factors (VIF). Those with a VIF below two were included in the final model at multiple logistic regression. The significant level was at less than 5% for a 95% confidence interval.

## Results

A total of 3104 records were retrieved. Of these, 1,068 had a diagnosis of BAD, with 83 (7.8%) having absconded from the hospital. Absconding was statistically more among those who did not receive psychotherapy than those who received (10.4% vs. 4.6%, X^2^ = 12.78, *p* < 0.001). Those who absconded had statistically fewer days on ward than those who did not, i.e., median of 4 vs. 7, respectively, *p* < 0.001. More participants with mood lability absconded than those without (13.2% vs. 7.3%, X^2^ = 4.06, *p*-value 0.044). More participants who used cannabis absconded than those who did not (25% vs. 7.5%, X^2^ = 6.71, *p*-value 0.010). Fewer participants who had irritability absconded compared to those who did not (3.5% vs. 8.4%, X^2^ = 4.30, *p*-value 0.038). Fewer participants were treated with haloperidol absconded than those who did not (3.6% vs. 8.8%, X^2^ = 6.45, *p*-value 0.011). Table 1.

**Table 1:** Participant characteristics distribution across absconding from hospital among patients with Bipolar Affective Disorder

Variable	Escape		X^2^ (p-value)
	**No** (985/ 92.3%)	**Yes** (83/ 7.8%)	
Age (median, *IQR)*	29 (23–40)	29 (24–38)	(0.922)
Marital status			
Divorced	11 (91.7)	1 (8.3)	1.20 (0.878)
Married	366 (94.3)	22 (5.7)	
Separated	61 (92.4)	5 (7.6)	
Single	345 (92.5)	28 (7.5)	
Widowed	25 (92.6)	2 (7.4)	
Gender			
Female	506 (93.7)	34 (6.3)	3.38 (0.066)
Male	477 (90.7)	49 (9.3)	
Employment status			
Unemployed	652 (91.8)	58 (8.2)	0.03 (0.873)
Employed	119 (92.3)	10 (7.7)	
Positive family history of mental illness			
No	765 (92.6)	61 (7.4)	0.41 (0.520)
Yes	211 (91.3)	20 (8.7)	
Clinical presentation			
1. Hallucinations			
No	771 (92.0)	67 (8.0)	0.25 (0.614)
Yes	213 (93.0)	16 (7.0)	
2. Delusions			
No	837 (92.4)	69 (7.6)	0.22 (0.637)
Yes	147 (91.3)	14 (8.7)	
3. Uncoordinated speech			
No	749 (92.5)	61 (7.5)	0.29 (0.591)
Yes	235 (91.4)	22 (8.6)	
4. Suspiciousness			
No	938 (92.2)	79 (7.8)	0.00 (0.952)
Yes	46 (92.0)	4 (8.0)	
5. Poor sleep			
No	342 (90.5)	36 (9.5)	2.49 (0.115)
Yes	642 (93.2)	47 (6.8)	
6. Aggression			
No	493 (93.2)	36 (6.8)	1.39 (0.239)
Yes	491 (91.3)	47 (8.7)	
7. Food refusal			
No	902 (91.8)	81 (8.2)	3.70 (0.054)
Yes	82 (97.6)	2 (2.4)	
8. Affective lability			
No	905 (92.7)	71 (7.3)	**4.06 (0.044)**
Yes	79 (86.8)	12 (13.2)	
9. Talking to self			
No	902 (92.2)	76 (7.8)	0.00 (0.975)
Yes	82 (92.1)	7 (7.9)	
10. Restlessness			
No	634 (91.7)	57 (8.3)	0.604 (0.437)
Yes	350 (93.1)	26 (6.9)	
11. Neglect of body hygiene			
No	890 (92.5)	72 (7.5)	1.18 (0.277)
Yes	94 (89.5)	11 (10.5)	
12. Destructiveness			
No	790 (92.8)	61 (7.2)	2.19 (0.139)
Yes	194 (89.8)	22 (10.2)	
13. Singing			
No	906 (92.3)	76 (7.7)	0.03 (0.870)
Yes	78 (91.8)	7 (8.2)	
14. Prayerfulness/ religiosity			
No	929 (92.2)	79 (7.8)	0.09 (0.768)
Yes	55 (93.2)	4 (6.8)	
15. Excessive energy			
No	681 (92.0)	59 (8.0)	0.13 (0.722)
Yes	303 (92.7)	24 (7.3)	
16. Wandering away from home			
No	748 (92.8)	58 (7.2)	1.56 (0.212)
Yes	236 (90.4)	25 (9.6)	
17. Talkativeness			
No	366 (92.9)	28 (7.1)	0.39 (0.530)
Yes	618 (91.8)	55 (8.2)	
18. Social withdrawal			
No	954 (92.1)	82 (7.9)	0.92 (0.337)
Yes	30 (96.8)	1 (3.2)	
19. Suicidal ideations			
No	954 (92.1)	82 (7.9)	0.92 (0.337)
Yes	30 (96.8)	1 (3.2)	
20. Grandiosity			
No	892 (92.2)	75 (7.8)	0.01 (0.931)
Yes	92 (92.0)	8 (8.0)	
21. Thoughtlessness			
No	975 (92.2)	83 (7.8)	0.77 (0.382)
Yes	9 (100)	-	
22. Poor appetite			
No	915 (92.0)	80 (8.0)	1.40 (0.236)
Yes	69 (95.8)	3 (4.2)	
23. Undressing			
No	927 (92.6)	74 (7.4)	3.36 (0.067)
Yes	57 (86.4)	9 (13.6)	
24. Irritability			
No	845 (91.6)	78 (8.4)	**4.30 (0.038)**
Yes	139 (96.5)	5 (3.5)	
25. Disorganized behavior			
No	927 (92.1)	80 (7.9)	0.68 (0.408)
Yes	57 (95.0)	3 (5.0)	
Current comorbid substance use			
1. Use of Cannabis			
No	972 (92.5)	79 (7.5)	**6.71 (0.010)**
Yes	12 (75.0)	4 (25.0)	
2. Use of alcohol			
No	977 (92.3)	82 (7.7)	0.14 (0.707)
Yes	8 (88.9)	1 (11.1)	
3. Multiple substance use			
No	974 (92.2)	82 (7.8)	0.03 (0.870)
Yes	10 (90.9)	1 (9.1)	
Days with symptoms before admission (median, IQR)	7 (3–14)	7 (4–14)	(0.3592)
Days of admission (median, IQR)	7 (4–12)	4 (2–9)	**(< 0.001)**
Current in-patient treatment			
Psychotherapy			
No	522 (89.5)	61 (10.4)	**12.78 (< 0.001)**
Yes	460 (95.4)	22 (4.6)	
Chlorpromazine			
No	117 (95.9)	5 (4.1)	2.55 (0.110)
Yes	850 (91.8)	76 (8.2)	
Haloperidol			
No	756 (91.2)	73 (8.8)	**6.45 (0.011)**
Yes	211 (96.4)	8 (3.6)	
Risperidone			
No	946 (92.1)	81 (7.9)	1.79 (0.180)
Yes	21 (100)	-	
Olanzapine			
No	964 (92.3)	80 (7.7)	1.68 (0.195)
Yes	3 (75.0)	1 (25.0)	
Trifluoperazine			
No	940 (92.3)	79 (7.8)	0.03 (0.865)
Yes	27 (93.1)	2 (6.9)	
Fluphenazine			
No	874 (92.1)	75 (7.9)	0.43 (0.514)
Yes	93 (93.9)	6 (6.1)	
Quetiapine			
No	958 (92.3)	80 (7.7)	0.07 (0.787)
Yes	9 (90.0)	1 (10.0)	
Carbamazepine			
No	43 (91.5)	4 (8.5)	0.04 (0.846)
Yes	776 (92.3)	65 (7.7)	
Lithium			
No	812 (92.3)	68 (7.7)	0.25 (0.616)
Yes	7 (87.5)	1 (12.5)	
Sodium valproate			
No	767 (92.1)	66 (7.9)	0.44 (0.508)
Yes	52 (94.5)	3 (5.5)	

### Factors associated with absconding from the hospital

The values significant at inferential statistics, i.e., chi-square and rank sum test, were included in logistic analysis. All variables with a *p*-value of less than 0.2 were tested for collinearity before being included in the final model. The likelihood of absconding among those with BAD increased with current use of cannabis [adjusted odds ratio (aOR) = 4.00, 95% confidence interval (CI) = 1.22–13.09, p-value = 0.022] and having mood lability [aOR = 2.15, 95 CI = 1.10–4.21, p-value = 0.025]. However, having psychotherapy (aOR = 0.44, 95 CI = 0.26–0.74, p-value = 0.002) and treatment with haloperidol (aOR = 0.39, 95% CI = 0.18–0.83, p-value = 0.014) reduced the likelihood of absconding. Table 2.

**Table 2:** Logistic analysis for factors associated with absconding from hospital among patients with Bipolar Affective Disorder

Variable	Bi variable analysis		Multivariable analysis	
	Crude Odds ratio (95% confidence interval)	p-value	Adjusted odds ratio (95% confidence interval)	P-value
**Number of days admitted**	0.99 (0.97–1.02)	0.724	**-**	
**Affective lability**				
No	1 (reference)		1 (reference)	
Yes	1.94 (0.01–3.72)	0.048	2.15 (1.10–4.21)	**0.025**
**Current comorbid use of cannabis**				
No	1 (reference)		1 (reference)	
Yes	4.10 (1.29–13.01)	0.017	4.00 (1.22–13.09)	**0.022**
**Irritability**				
No	1 (reference)		1 (reference)	
Yes	0.039 (0.16–0.98)	0.045	0.38 (0.13–1.06)	0.064
**Psychotherapy**				
No	1 (reference)		1 (reference)	
Yes	0.41 (0.25–0.68)	0.001	0.44 (0.26–0.74)	**0.002**
**In-patient Haloperidol treatment**				
No	1 (reference)		1 (reference)	
Yes	0.39 (0.19–0.83)	0.014	0.39 (0.18–0.83)	**0.014**

## Discussion

The study aimed determine the prevalence and associated factors of absconding from the hospital following admission among individuals with BAD using a retrospective chart review of the inpatient from January 2018 to December 2021 at Mbarara Regional Referral psychiatry ward.

The prevalence of absconding across the entire study period was 7.8%, a percentage similar to that found among psychiatric patients of various diagnoses in South Africa of 7.83 [19]. However, the prevalence observed in this study was much lower than that of 45.4% seen among participants with BAD in a two months prospective study in India [16]. This difference could be due to the significant difference in the study duration, study design, study setting and probably because the study by Khisty et al. (2008) had a small overall sample size of participants with BAD of 63 [16]. Similarly, the prevalence observed in this study was lower than that of 21.4% among those with BAD in a one year prospective study in Iran [23]. This difference could also be due to the significant difference in the study duration, study design, study setting and probably because the study by Sheikhmoonesi et al. (2012) had a small overall sample size of participants with BAD of 12 [23].

The likelihood of absconding increased with having affective lability, a finding congruent with that of Andoh et al. (2004), who discovered that having a very labile mood is strong associated with absconding from mental health facilities [24]. Patients with affective lability are most likely have exaggerated impairments in inhibitory control due to exaggerated activation in several regions of the Prefrontal cortex (PFC) as well as the anterior cingulate gyrus and caudate by the over stimulated amygdala from excess serotonin to this region [25–27]. This makes them prone to making impulsive decisions such as absconding. Additionally, affective lability is associated with greater severity in the clinical expression of BAD with more frequent comorbidities of anxiety disorders and substance use disorders [28], that are likely to cause impairment in decision making. It is possible that patients with affective lability may abscond from hospitals due to other factors, including a lack of understanding of their condition, frustration with the hospital environment or treatment, or a desire to escape from the perceived constraints or structure of the hospital setting [18].

Comorbid use of cannabis among participants with BAD increased the likelihood of absconding. This finding was congruent with that by Wilkie et al. (2014) who reported that comorbid substance use almost triples the odds of absconding among forensic patients [29]. The explanation for this finding is most likely because substances like cannabis use causes brain atrophy impairing one’s cognition and executive functioning [30], worsening decision-making, increasing impulsive acts, and further complicating the course of BAD with a worsening prognosis [31]. In addition, the following reasons may lead to the increased likelihood of absconding of patients with BAD who use cannabis. (i) Lack of understanding: Some people may not fully understand the risks associated with using cannabis while receiving treatment for a mental health condition like bipolar disorder. They may feel that the cannabis is helping them feel better and are unaware of the potential negative effects on their treatment. (ii) Relief of symptoms: Cannabis can potentially help to reduce anxiety and improve mood [32–34], which may make someone feel better in the short term. This could lead them to believe that they no longer need treatment and they may abscond from the hospital. (iii) Difficulty with treatment: Being in a hospital can be overwhelming and stressful, particularly if someone is not used to being in a hospital setting [35]. This stress, combined with the symptoms of bipolar disorder, could make someone feel like they want to leave and find relief elsewhere, potentially leading to absconding. It is important to emphasize to patients not to use cannabis while receiving treatment for bipolar disorder, as it can interfere with the effectiveness of treatment and potentially worsen symptoms in the long term [36–38].

Having a current in-patient treatment with haloperidol was protective against absconding in participants with BAD. This finding is congruent with studies that point out the protective role of haloperidol in challenging behavior, an example of which is absconding, among mental illness patients [39, 40]. This is likely because manic symptoms of BAD, like excitation, irritability, and elevated mood, which are more likely to increase absconding tendencies, are possibly due to elevated levels of extracellular Dopamine (DA) in areas receiving dopaminergic innervation, with the consequent activation of DA receptors [41–43]. Haloperidol is a high-potent DA antagonist [44], alleviating manic symptoms of BAD. In addition, haloperidol, compared to other antipsychotics in the present study is associated with more extrapyramidal side effects associated with disturbances in mobility and motivation [45], which could interfere with the desire to abscond. However, the present study did not capture information regarding the dosage, route of administration, and frequent of use, that may influence the pharmacodynamics of the medications.

Psychotherapy was also protective against absconding. This finding is congruent with various studies that state the role of psychotherapy in alleviating absconding among those with mental illness [3, 46, 47]. This is likely because the first step of the intervention involving effective educational interventions of psychotherapy, like description of the problem to be treated, its causes if known, and the methods of intervention to be used to alleviate the problem, is a predictor of better outcomes of interventions for psychiatric disorders [48]. This can make them feel more in control of their lives and more able to cope with their challenges. Psychotherapy can also provide a safe and supportive environment where individuals can discuss their thoughts and feelings without fear of judgment [49, 50]. This can help them feel more connected and less isolated, which may reduce the likelihood of them wanting to leave treatment. In addition, psychotherapy can help individuals develop healthy coping skills and strategies for managing their symptoms [50, 51]. This can improve their overall well-being and reduce the need for hospitalization or other forms of treatment. It is important to note that psychotherapy is just one part of a comprehensive treatment plan for bipolar disorder and may be used in combination with medication and other therapies. With multiple factors involved in absconding, holistic modalities and care plans should be emphasized in dealing with absconding.

## Limitations

This study’s findings should be interpreted with caution in view of the following limitations. First, this was a retrospective chart review, and missing data are not uncommon in such study designs. To overcome this issue, we recommend future researchers to make provisions for multiple assessment methods, such as prospective study designs. Second, absconding was reported in the participant’s charts by nurses who are less likely to report those missing if risks involved are perceived as very low or absconded after they were observed as recovered. We recommend the using standard operational criteria to prevent absconding. In addition, the symptoms reported such as irritability, affective lability, and among others, were based on information captured by the experienced clinicians at the psychiatric facility. We recommend the use of clinically validated tools to measure and capture symptoms in future studies. Thirdly, many variables that could have influenced the findings, such as time of the day when participants absconded and financial constraints as regards to feeding and medication unavailability on ward, as previously described by Kaggwa et al. (2021) [18]; were not retrieved from the records of patients and hence not included in the analysis. We recommend future studies to use prospective study designs to capture such information. Our study did not specify the type of psychotherapy that was effective in reducing absconding. We recommend future researchers to investigate the different psychotherapies for better recommendations. Also, the sample size of the study was small, and this may not be statistically powered enough. We recommend large sample studies involving multiple sites to ensure generalizability and statistical accuracy. Finally, the data included in the analysis were from one facility, limiting the generalization of the findings throughout the country. Although the sample of patients with BAD in our study cannot be said to be representative of Uganda as a whole, it could be assumed that the estimate could have been higher considering that among those who receive orthodox clinical attention, only a fraction come to tertiary health facilities where the present study was conducted. Some patients may have presented to primary health facilities, secondary health facilities or other private health facilities.

## Conclusions

In this retrospective review of cases of absconding among those with BAD over a four-year period in Uganda, it can be concluded that absconding among patients with BAD is common in Uganda. Those with symptoms of affective lability and those with comorbid cannabis use tend to abscond more while those who receive haloperidol and psychotherapy are less likely to abscond. Further studies looking at absconding among those with BAD and other psychiatric conditions using a prospective approach are warranted to provide extended causal relationships and management strategies.

## Data Availability

The datasets will be made available to appropriate academic parties on request from the corresponding author.
